# The COVID-19 pandemic and self-reported food insecurity among women in Burkina Faso: evidence from the performance monitoring for action (PMA) COVID-19 survey data

**DOI:** 10.1186/s12905-025-03565-x

**Published:** 2025-02-01

**Authors:** Ortis Yankey, Marcellinus Essah, Prince M. Amegbor

**Affiliations:** 1 School of Geography and Environmental Science, WorldPop Research Group, University of Southampton, University Road, Southampton, SO17 1BJ UK; 2 Department of Geography and Planning, University of Toronto, Sidney Smith Hall, 100 St. George Street, Toronto, ON M5S 3G3 Canada; 3 New York University School of Global Public Health, New York, USA

**Keywords:** Food insecurity, COVID-19, Women’s food insecurity, Burkina Faso, Agricultural policies, Performance monitoring for Action (PMA)

## Abstract

**Background:**

The COVID-19 pandemic led to widespread economic disruptions, with government-imposed restrictions and lockdowns significantly affecting livelihoods globally. Burkina Faso, a country with pre-existing vulnerabilities in food security, experienced considerable challenges during this period. The aim of this study was to examine how COVID-19-related income losses is associated with self-reported food insecurity among women in Burkina Faso in 2020. The study also examined whether there was an increase in self-reported food insecurity among women during the COVID-19 restrictions compared with the pre-pandemic era.

**Methods:**

We conducted a cross-sectional analysis using data from the Performance Monitoring for Action (PMA) female survey, which included 3,499 women from Burkina Faso. This study examined the associations between socioeconomic variables, such as age, education, household income loss, and food insecurity. We conducted two analyses using logistic regression. The first analysis focused on self-reported food insecurity and its association with the socioeconomic variables, and the second analysis focused on whether there was an increase in self-reported food insecurity compared with pre-pandemic levels and its association with the socioeconomic factors. We controlled for relevant confounders in the analysis and presented the results as adjusted odds ratios (AORs) with 95% confidence intervals (CIs).

**Results:**

Our findings indicated that 16.97% of women reported experiencing food insecurity during the pandemic period. Compared with women with no income loss, women who experienced partial household income loss were 1.82 times (95% CI: 0.98–3.38) more likely to report food insecurity, whereas those who experienced complete income loss were 5.16 times (95% CI: 2.28–9.43) more likely to report food insecurity. The study, however, did not find a statistically significant increase in self-reported food insecurity due to COVID-19 restrictions compared with pre-pandemic levels.

**Conclusions:**

This study demonstrated that income loss due to COVID-19 restrictions profoundly affected women’s food security in Burkina Faso. The significant associations between income loss and increased food insecurity underscore the need for targeted interventions and safety nets to support women during public health crises.

## Introduction

The COVID-19 pandemic in 2020 threatened the food security status of many poor and vulnerable people worldwide, especially in developing countries. According to the United Nations Food and Agriculture Organization (FAO), food security exists “when all people, at all times, have physical, social, and economic access to sufficient, safe, and nutritious food that meets their dietary needs and food preferences for an active and healthy life” [12]. Therefore, any phenomenon that tends to disrupt people’s ability to meet their dietary needs and food preferences for an active and healthy life is more likely to render people food insecure. The COVID-19 pandemic continues to undermine the food security status and livelihoods of many people around the world. In 2020, the United Nations estimated that more than a quarter of a billion people could face starvation due to the COVID-19 pandemic [41].

While the COVID-19 pandemic has caused devastating impacts globally, countries such as Burkina Faso have experienced the worst impacts because of the combined issues of climate change and food insecurity. Burkina Faso reported its first COVID-19 case on March 9, 2020, and approximately nine [7] out of its thirteen (13) regions had reported COVID-19 cases by December 2020 [30]. Since the onset of the COVID-19 pandemic in Burkina Faso, households have lost their livelihoods due to the loss of income and high levels of malnutrition. For example, Zidouemba et al. [49] reported that the COVID-19 pandemic has aggravated the food insecurity situation among rural and urban households in Burkina Faso. Furthermore, the World Food Program (WFP) reported in August 2020 that more than 3 million people in Burkina Faso faced acute food insecurity as they battled with COVID-19 and conflict [46]. These dynamics continue to threaten Burkina Faso’s ability to achieve Sustainable Development Goal 2 (Zero Hunger), particularly for women. Because women contribute immensely to providing care and frontline healthcare during epidemics and pandemics, they disproportionately face the burden of global health pandemics [9, 18]. Indeed, in Nairobi, it was recorded that women spent five times more time on unpaid care work than men did during global health outbreaks [32]. Additionally, Davies and Bennet [8] documented how the outbreak of the Ebola virus in Africa placed an enormous burden on female caregivers and front-line workers.

While several studies have examined the effects of global health outbreaks on women, there is limited knowledge about how COVID-19 restrictions have specifically impacted the income levels and food security status of women worldwide. A systematic review by Vasseur et al. [43] revealed that the experiences of women were often missing from discussions about food security during the pandemic. This lack of attention to women’s experiences extends to food security research and contributes to the lack of focus on women in food and nutrition policies globally [44]. Our paper fills a knowledge gap concerning women and food insecurity during the COVID-19 pandemic. Understanding the effects of the COVID-19 pandemic on the income levels of women in Burkina Faso is critical to understanding how the global health pandemic may affect women and render them vulnerable to food insecurity. Therefore, our paper draws on the COVID-19 data from the Performance Monitoring for Action (PMA) female survey to:

1. Determine whether the loss of income due to the COVID-19 restrictions in Burkina Faso is associated with self-reported food insecurity among Burkinabe women aged 15–49 years.
2. Examine whether self-reported food insecurity increased due to the COVID-19 restrictions compared with pre-pandemic period.

The rest of the paper is organized as follows. Section two sheds light on the concept of vulnerability and entitlements, the framework for examining the issues under study. Section three presents the study area, methods, and results of the study. Section four focuses on the discussion of the results, limitations, and conclusions.

### The vulnerability framework and entitlements approach

We draw from the concepts of Vulnerability and Entitlements to illuminate the vulnerability of the global food system to the COVID-19 pandemic and to explore the experiences of women in Burkina Faso during the COVID-19 pandemic. We focus on how the loss of income due to COVID-19 restrictions impacted women’s vulnerability to food insecurity in Burkina Faso. Wisner et al. ( [47]:11) defined vulnerability as the characteristics of a person or group and their situation that influence their capacity to anticipate, cope with, resist, and recover from the impact of a natural hazard (an extreme natural event or process). They noted that different people possess different vulnerabilities during hazards, with some being more prone to damage, loss, and suffering. The determining variables include class, occupation, immigration status, caste, ethnicity, gender, disability and health status, age, and the nature and extent of the social network.

Similarly, Chambers [6] argued that vulnerability has two sides: an external side of risk, shock, and stress to which an individual or household is subject; and an internal side of defenselessness, meaning a lack of means to cope without damaging loss. Despite the two sides of vulnerability, Moseley and Battersby [26] also noted that the vulnerability of African food systems is underpinned by the location of the country and the position of the country in the global political economy. In this paper, we use the term “vulnerability” to refer to people who are at the worst end of the pandemic and are unlikely to rebuild their livelihoods [47]. In doing so, we focus on the social constructivist approach to vulnerability.

The social constructivist approach to vulnerability classifies vulnerability as a social process, inherent or entrenched in power relations and historical patterns of inequalities that continue to exist in society [34]. Indeed, knowledge from existing research has shown that traditional gender norms and socioeconomic policies that favour men, contribute to women’s vulnerability and low economic outputs in sub-Saharan Africa [22]. We situate vulnerability as a combination of three factors: the probability of exposure, the degree to which a population would be impacted by an outbreak, and the ability of a population to recover from the disease. These three factors are key to our paper because the COVID-19 pandemic has exposed vulnerabilities in the global food system, and some countries may not be able to recover from the impacts of the pandemic. Notwithstanding, while the probability of exposure, the degree to which a population would be impacted by an outbreak, and the ability of a population to recover from the disease draw our attention to the COVID-19 exposure and food (in)security in Burkina Faso, we also acknowledge that ‘access’ to resources, in this case, food, is paramount during crises; hence, we rely on the Entitlement Approach to show how access to resources played a role in the lives of women during the COVID-19 pandemic.

Sen ( [37]:434) stated that “the entitlement approach concentrates on each person’s entitlements to commodity boundaries including food, and views starvation as resulting from a failure to be entitled to any bundles with enough food”. Thus, entitlement is shaped by two elements: personal endowments – the resources one has, such as livestock, land, and non-tangible goods – and the set of commodities a person has access to through trade and production, that is, ‘the exchange entitlement mapping’ ( [37]:435). In Sen’s view, when an individual has ownership rights and endowment of resources, they are likely to possess the capacity to acquire sufficient resources when there is scarcity of resources. Indeed, Sen argued that the root causes of famine cannot be limited to the decline in food availability, rather, it is the outcome of entitlement failures – access. These entitlements hinge on questions of ownership, and are classified as the trade-based entitlement, production-based entitlement, labour-based entitlement, inheritance and transfer entitlement. Production, trade, and labour-based entitlements are mediated by wage labour and private property relations [36]. While these forms of entitlements define food access, levels of food accessibility differ by social groups including the economic situation of smallholder farmers, landholders, urban and rural labourers, migrant labourers, and along demographic lines such as gender and age. Reflecting this, Sen assert that shareholding arrangements provide people with a better accessibility to food compared to those in rural or urban areas who depend on wages during food price hikes [36].

Demographically, food access is a major issue in Burkina Faso for women. While women dominate the agricultural sector, they have unequal access to land, agricultural inputs, education, extension services and the market [21]. In addition, women have an enormous labour burden and care work at home. To address these issues, in 2009, the government of Burkina Faso adopted the National Policy for Securing Landownership to ensure an equitable access to land and equitable use of land. In that same year, the government adopted the National Gender Policy to address and promote gender equality [28]. While Sen’s argument likely explains famine at the individual level, other scholars have argued that the focus must shift from individual entitlements to broadening the scope to issues such as collective action [14], the influence of social structures [19], and a reduction in market failures, which are likely to lead to new endowments [38].

## Methodology

### Study context

As a landlocked country in West Africa, Burkina Faso has a population growth rate of approximately 2.8%, with a current population figure approximately 20.9 million [42]. The population growth in urban areas has remained high over the years, ranging from 4.9 − 6.7% from 2009 to 2019. About 80% of the population in Burkina Faso is dependent on agriculture, and more than 20% of the population is food insecure; 40.1% of the population remains below the poverty line, and an additional 86% of the population still relies on subsistence agriculture [42]. In rural Burkina Faso, subsistence agriculture is the mainstay of the economy. In fact, most rural families are known to be poor and have children at risk of infectious diseases and malnutrition. The ongoing insecurity situation, food price inflation, climate change, and the residual effects of the COVID-19 crisis continue to worsen food insecurity in the country. For example, in the western part of Burkina Faso, increasing insecurity and production deficits have led to households resorting to one meal a day, including decreasing the size and quality of food [13].

### Data source

Data for this study were obtained from the Performance Monitoring for Action (PMA) female survey (https://www.pmadata.org/). The PMA conducts frequent and high-quality surveys to track key health indicators in ten African and Asian countries for women aged 15–49 years. PMA data largely focus on family planning, and the data are collected on a repeated basis, usually within a six-week period based on enumeration areas [50]. The PMA data are de-identified, and the data provide nationally representative information on family planning among women. The PMA uses a multi-stage cluster design in which urban and rural areas and major regions are used as strata in a given country. Enumeration areas within each stratum that provide a nationally representative sample are systematically selected for household surveys via random selection. Female respondents are the focus of these surveys, with a series of questions for all women of reproductive age [13, 40] living in each household (https://www.pmadata.org/).

While the focus of the PMA’s female surveys is on family planning, with the onset of COVID-19, the PMA also collected data on women’s COVID-19 experiences. For this study, we used the Burkina Faso Phase 1 COVID-19 female survey. This survey was conducted in June 2020 by telephone following a prior PMA household survey. The number of eligible respondents for the survey was 4691 women, out of whom 21.5% were not reached. A total of 95.8% of the remaining respondents completed the COVID-19 survey, yielding a response rate of 95.2% among eligible women [10]. The survey recorded the demographic profile of the women, COVID-19 related experiences, COVID-19 information sources, COVID-19 knowledge, COVID-19 prevention, and perceptions of COVID-19. The survey was conducted in 167 enumeration areas in Burkina Faso. The data also included a survey weight, which was used to adjust for differences in the probabilities of selection and response rates of the women in the sample to ensure that the results obtained from the survey were representative of the target population.

### Measurement - outcome variables

Our study conducted two separate analyses to explore food insecurity experiences during the COVID-19 pandemic. In the first analysis, we examined women’s experiences of food insecurity during the pandemic and their associations with socioeconomic factors. The outcome variable for this analysis was food insecurity, measured by the following question:

1. Since the coronavirus (COVID-19) restrictions began, did you or any household member go a whole day and night without eating anything because there was not enough food? This question had four responses. (i) Yes (ii) No (iii) Unsure (iv) No response.

**Fig. 1:**
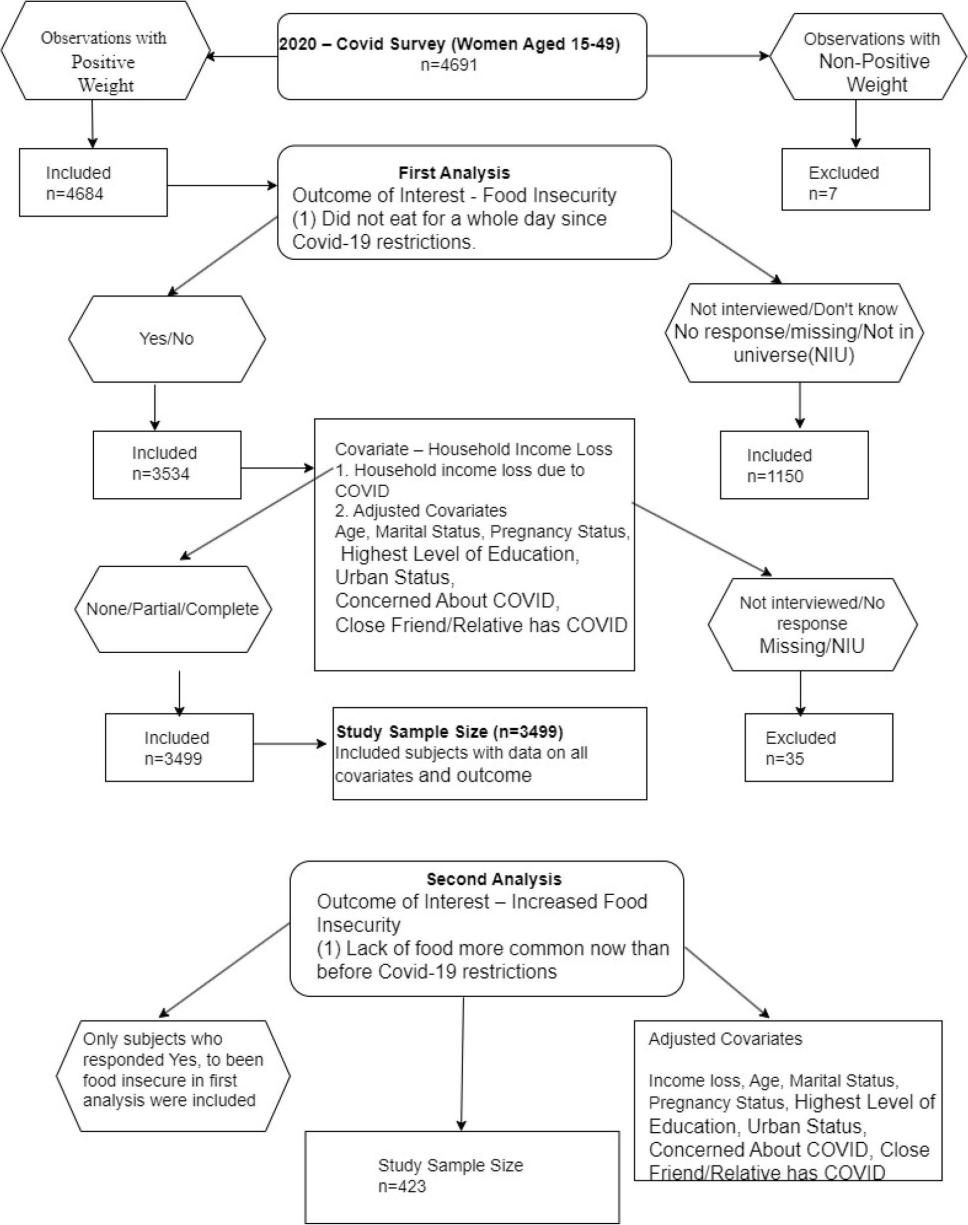
Data processing workflow

We classified respondents who answered “Yes” as food insecure, and those who responded “No” as food secure. Respondents who refused to answer or were unsure were excluded from the analysis. This led to the inclusion of 3499 individuals in this analysis (see Fig. 1). Our dependent variable for this analysis was food insecurity, which had two values (Yes or No).

In the second analysis, we explored whether there was an increase in self-reported food insecurity compared with that in the pre-COVID-19 period. This was measured using the following question:

2. Is a lack of food more common now compared to before the coronavirus (COVID-19) restrictions began?
3. This question had two responses: (i) Yes, (ii) No.

The respondents who answered “Yes” were classified as experiencing an increase in food insecurity due to COVID-19. Thus, our dependent variable in this second analysis is increase in self-reported food insecurity, which also had two values (Yes or No).

Importantly, the second question was administered only to respondents who had already been classified as food insecure based on the first question. Therefore, only those who had gone a whole day and night without food during the pandemic were asked whether food insecurity had increased compared with pre-pandemic times. This resulted in a subpopulation of 423 respondents for the second analysis (see Fig. 1).

### Explanatory variables (independent variables)

COVID-19 related experiences and socioeconomic covariates were used as explanatory variables in the study. These variables were household income loss due to COVID-19 restrictions (none, partial, complete), age, marital status (single, married, divorced/separated, widowed), pregnancy status (yes or no), highest level of education (never attended school, primary/middle school, secondary/post-primary, tertiary/post -secondary), urban status (yes or no), concerned about COVID-19 (not concerned, a little concerned, and very concerned) and whether a close relative/friend has COVID-19 (yes or no). Variables that had missing values or “no response” were excluded from the data. We used these variables as independent variables in both analyses specified above.

These variables were chosen because they are known to influence economic stability, access to resources, and health outcomes, which are key factors affecting food insecurity [, 20, 29]. For instance, income loss directly impacts a household’s ability to purchase food, whereas education level and urban status are often linked to access to social safety nets and resources. COVID-19-related factors such as concern about the virus or having a close contact with the illness may also affect food security through disruptions in social and economic networks. Thus, these covariates selected for analysis in this study were influenced by theory, the literature on food insecurity, data availability, and prior research on food insecurity and pandemics.

### Statistical analysis

The sampling weights provided by the PMA data, which were adjusted for follow-up loss and survey non-response, were used to account for the complex sampling design and to obtain population-based estimates for Burkina Faso. To describe the characteristics of the study population, weighted prevalence estimates and corresponding 95% confidence intervals (CIs) were computed based on the sample of individuals (*n* = 3499 for the first outcome, and *n* = 423 for the second outcome), with complete data for all variables considered in this study, as provided in (Fig. 1).

A logistic regression model was used to examine the associations between the explanatory variables and the outcome variables (food insecurity and increase in food insecurity) in two separate analyses. The observed outcomes are denoted as $\:{y}_{i}^{\left(m\right)}$ where $\:m$ is either food insecurity or an increase in food insecurity is binary (yes or no) and is given as

$$\:{y}_{i}^{\left(m\right)}=\begin{array}{c}1=\text{Y}\text{e}\text{s}\:\:\\\:0=No\:\end{array}$$

Because the outcome variables are categorical, a logit link function was used to express the set of covariates given as $\:{\:X}_{n}$as a function of the outcome variables. The conditional probability that the *i*^th^ respondent was food insecure or experienced increased food insecurity given the set of independent variables $\:{X}_{i}$ is denoted by $\:{P}_{i}=P({y}_{i}=1|{X}_{i})$. The model is expressed in a linear form and given as :

$$\:{logit(P}_{i})=\text{ln}\left(\frac{{\:P}_{i}}{1-{\:P}_{i}}\right)\:={\beta\:}_{0}+{\beta\:}_{1}{X}_{i1}+{\beta\:}_{2}{X}_{i2}+\dots\:+{\beta\:}_{n}{X}_{in}\:,\:i=\text{1,2},\dots\:,n$$

where$\:\:\beta\:$’s are the regression coefficients for the covariates. All analyses were conducted in SAS 9.3 (SAS Institute, Cary, NC, USA) using SAS survey procedures (PROC SURVEYFREQ, PROC SURVEYMEANS, PROC SURVEYLOGISTIC). These procedures were employed specifically to account for the complex, multi-stage sampling design of the survey. This ensured that the standard errors and confidence intervals were adjusted accordingly, reflecting the study’s sampling approach.

## Results

### Participant characteristics

Table 1 shows the overall characteristics of the study participants, as well as the weighted proportions of the study variables and their respective 95% confidence intervals (95% CI). The prevalence of food insecurity among women aged 15–49 years during the COVID-19 restrictions was 16.97% (95% CI: 13.32–20.62). About 26% (95% CI: 20.70–31.21) reported no income loss, 58.33% (95% CI: 52.98.−63.68) reported a partial income loss, and 15.72% (95% CI: 10.67–20.77) reported a complete income loss due to the COVID-19 restrictions. The average age of the women was 27.91 years old (95% CI 27.12–28.71). Most of these women were married (74.95%) and were not pregnant during the time of the survey (91.93%). More than half of these women (54.85%) never attended school, and the majority resided in rural areas (78.54%). Most of the women (66.96%) reported that they were very concerned about COVID-19. However, only about 1.63% (95% CI: 0.47–2.78) of these women had relatives or friends who had contracted COVID-19.

Table 1 also reports the prevalence of self-reported food insecurity (yes) and the characteristics of the participants. Among the women, 34.32% (95% CI: 27.55–41.09) of the individuals who lost complete household income reported being food insecure during the COVID-19 restrictions. This percentage was relatively greater than that of individuals who did not lose income (10.05%, 95% CI: 3.96–16.13) and individuals who lost partial income (15.38%, 95% CI: 11.33–19.42). A greater proportion of married women (18.50%, 95% CI: 14.37–22.62) reported being food insecure during the lockdown than other women who were single, divorced or widowed. Food insecurity was also relatively higher among pregnant women (19.71%, 95% CI: 10.42–28.99), women who never attended school (20.19%, 95% CI: 15.44–24.95), women who were living in rural areas (18.68%, 95% CI: 14.29–23.06), and women who reported that a family member or friend had COVID-19 (51.7%, 95% CI: 16.20–87.20).

**Table 1:** Descriptive statistics of participant (women aged 15 −49years, *n* = 3499)

				*Food Insecurity Status (Yes)*	
	Unweighted n	Proportion (%)	95% CI	Proportion (%)	95% CI
**Food Insecurity Status**					
No	3076	83.03	79.38–86.68		
Yes	423	16.97	13.32–20.62		
**Household Income Loss**					
None	813	25.95	20.70–31.21	10.05	3.96–16.13
Partial	2137	58.33	52.98–63.68	15.38	11.33–19.42
Complete	549	15.72	10.67–20.77	34.32	27.55–41.09
Socio-demographics					
**Age in years**	3499	27.91^a^	27.12–28.71	27.83^a^	26.15–29.50
**Marital Status**					
Single	876	21.80	17.04–26.56	11.25	4.07–18.43
Married	2422	74.95	69.67–80.23	18.50	14.37–22.62
Divorced/Separated	125	1.96	1.07–2.87	16.78	2.81–30.75
Widow	76	1.29	0.70–1.88	25.13	3.96–47.30
**Pregnancy Status**					
No	3228	91.93	90.43–93.42	16.73	12.98–20.48
Yes	271	8.07	6.58–9.57	19.71	10.42–28.99
**Education**					
Never Attended	1199	54.85	50.13–59.77	20.19	15.44–24.95
Primary/Middle School	698	19.30	15.13–23.46	15.77	7.64–23.91
Secondary/Post Primary	1365	24	19.76–28.25	11.44	6.26–16.63
Tertiary/Post-Secondary	237	1.84	1.29–2.38	5.39	1.48–9.30
**Urban Status**					
No	884	78.54	72.48–84.59	18.68	14.29–23.06
Yes	2615	21.46	15.41–27.52	10.71	9.06–12.36
**Concerned about COVID**					
Not concerned	147	4.11	2.58–5.65	3.83	1.27–6.39
A little concerned	367	7.44	5.39–9.50	18.91	0.51–37.32
Concerned	846	21.48	16.29–26.67	16.95	9.99–23.92
Very concerned	2139	66.96	60.33–73.59	17.57	13.22–21.92
**Friend/Family has Covid**					
No	3413	98.37	97.22–99.52	16.4	12.81–19.98
Yes	86	1.63	0.47–2.78	51.7	16.20–87.20

^a^Presented as Mean Value

### Associations between Self-reported Food Insecurity and the socioeconomic variables

Table 2 presents the unadjusted and adjusted effects of the explanatory variables on food insecurity. The unadjusted effect is a bivariate association between food insecurity and each independent variable that does not control for other covariates or confounders. The adjusted effect measures the overall association between the dependent variable (food security) and all the covariates in the study.

In terms of the unadjusted effect, we found that women who reported complete household income loss due to the COVID-19 restrictions were more than 4 times more likely to report food insecurity than were those who reported no household income loss (Unadjusted Odd Ratio, UOR = 4.67, 95% CI: 2.42–9.05). Women who reported losing partial household income were 1.63 times more likely to report food insecurity than those who reported no income loss (UOR = 1.63, 95% CI: 0.80–3.31). The unadjusted effect of education also revealed that women who had secondary and tertiary education were 0.51 times (95% CI: 0.29–0.91) and 0.23 times (95% CI: 0.10–0.50) less likely to report food insecurity, respectively, than women who never attended school. The results further indicate that living within an urban environment significantly decreases the odds of food insecurity among the women living in urban areas relative to women who were not living within an urban area (UOR = 0.52, 95% CI: 0.37–0.74). We also found from the unadjusted effect that women who were concerned about COVID-19 were 5 times more likely to report food insecurity relative to those who were not concerned (a little concerned UOR = 5.86, 95% CI: 1.42–24.15), (concerned UOR = 5.13, 95% CI: 2.27–11.59), and (very concerned UOR = 5.35, 95% CI: 2.66–10.78). Similarly, women who reported having a friend or family member infected with COVID were also more than 5 times more likely to experience food insecurity (UOR = 5.46, 95% CI: 1.28–23.34).

After all covariates of interest were adjusted, women who reported complete income loss were 5 times more likely to report food insecurity than those who reported no income loss (AOR = 5.16, 95% CI: 2.82–9.41). We also observed a 0.3-fold decrease in the odds of experiencing food insecurity for women who had tertiary education compared with those who had never attended school (AOR = 0.3, 95% CI: 0.10–0.88). Similarly, living within an urban area decreased the odds of experiencing food insecurity relative to women not living within an urban area (AOR = 0.59, 95% CI: 0.38–0.91). In contrast, women who were a little concerned about COVID-19, those who were concerned about COVID-19, and those who were very concerned about COVID-19 were more than 5 times, more than 4 times, and than 3 times more likely to report food insecurity, respectively. Similarly, women who reported that a friend or family member had contracted COVID-19 were more than 10 times more likely to report food insecurity (AOR = 10.94, 95% CI: 1.86–64.16).

**Table 2:** Association between Food Insecurity and the socioeconomic variables (*n* = 3449)

	Unadjusted Odds Ratio (UOR)	95% CI	Adjusted Odds Ratio (AOR)	95% CI
**Household Income Loss**				
None	Reference			
Partial	1.63	0.80–3.31	1.82	0.98–3.36
Complete	**4.67**^a^	**2.42–9.05**	**5.16**^a^	**2.83–9.41**
Socio-demographics				
**Age in years**	0.1	0.98–1.02	0.97	0.95–1.00
**Marital Status**				
Single	Reference			
Married	1.79	0.84–3.80	1.63	0.63–4.23
Divorced/Separated	1.59	0.48–5.29	1.95	0.60–6.31
Widow	2.65	0.61–11.53	3.43	0.54–21.90
**Pregnancy Status**				
No	Reference			
Yes	1.22	0.67–2.25	0.92	0.41–2.02
**Education**				
Never Attended	Reference			
Primary/Middle School	0.74	0.40–1.38	0.61	0.34–1.10
Secondary/Post Primary	**0.51**^a^	**0.29–0.91**	0.63	0.33–1.24
Tertiary/Post-Secondary	**0.23**^a^	**0.10–0.50**	**0.30**^a^	**0.10–0.88**
**Urban Status**				
No	Reference			
Yes	**0.52**^a^	**0.37–0.74**	**0.59**^a^	**0.38–0.91**
**Concerned About Covid**				
Not concerned	**Reference**			
A little concerned	**5.86**^a^	**1.42–24.15**	**5.77**^a^	**1.46–22.77**
Concerned	**5.13**^a^	**2.27–11.59**	**4.42**^a^	**1.73–11.30**
Very concerned	**5.35**^a^	**2.66–10.78**	**3.81**^a^	**1.76–8.25**
**Friend/Family has Covid**				
No	Reference		Reference	
Yes	**5.46**^a^	**1.28–23.34**	**10.94**^a^	**1.86–64.16**

^a^Statistical significance at 5% alpha

### Self-reported increase in food insecurity compared to pre-pandemic period

The women who reported experiencing food insecurity in the previous analysis were also asked whether the lack of food had become more common now (self-reported increase in food insecurity) than before the COVID-19 restrictions began. Self-reported increase in food insecurity was then examined against the socioeconomic variables to ascertain whether there was an association between the independent variables and the increase in self-reported food insecurity compared to pre-pandemic levels. Table 3 presents the results from the analysis.

In terms of the adjusted effect (Table 3), the odds of experiencing increased food insecurity were lower among the women who lost partial income and those who lost complete income than among women who lost no income. The odds of experiencing increased food insecurity were similarly lower among pregnant women, women who had some level of education, and women who had a family member or friend with COVID-19. In contrast, married women, divorced and widowed women, were more likely to experience increased hunger than single women were, and women who were concerned about COVID-19 were also more likely to experience increased food insecurity. However, it must be noted that none of the results in this analysis yielded a statistically significant result except for the unadjusted effect of women who were divorced or separated, which showed that they were 8 times more likely to experience frequent food insecurity than single women (UOR = 8.16, 95% CI: 1.42–47.35).

**Table 3:** Associationbetweenn Selfreporteddincreasee in Food Insecurity and thesocioeconomiccvariabless (*n* = 423)

	Unadjusted Odds Ratio (UOR) and 95% CI		Adjusted Odds Ratio (AOR) and 95% CI	
**Household Income Loss**				
None	Reference			
Partial	0.91	0.23–3.67	0.23	0.25–2.72
Complete	2.48	0.72–8.54	0.75	0.75–6.49
Socio-demographics				
**Age in years**	1.00	0.96–1.05	0.99	0.93–1.04
**Marital Status**				
Single	Reference			
Married	1.55	0.39–6.14	1.39	0.25–7.62
Divorced/Separated	**8.16**	**1.42–47.35**^a^	5.75	0.85–38.97
Widow	3.41	0.36–32.78	3.24	0.18–57.16
**Pregnancy Status**				
No	Reference			
Yes	0.60	0.16–2.23	0.54	0.13–2.26
**Education**				
Never Attended	Reference			
Primary/Middle School	0.91	0.35–2.34	0.92	0.41–2.07
Secondary/Post Primary	0.75	0.26–2.15	0.86	0.21–3.41
Tertiary/Post-Secondary	0.56	0.11–2.87	0.67	0.10–4.43
**Urban Status**				
No	Reference			
Yes	0.97	0.45–2.07	1.08	0.51–2.29
**Concerned About Covid**				
Not concerned	Reference			
A little concerned	1.76	0.27–11.74	2.82	0.30–26.33
Concerned	1.11	0.29–4.25	1.77	0.38–8.22
Very concerned	1.80	0.52–6.23	2.40	0.65–8.90
**Friend/Family has Covid**				
No	Reference		Reference	
Yes	0.50	0.05–4.96	0.60	0.06–6.41

^a^Indicate variables that have statistically significant associations at the p < 0.05 level in either the unadjusted or adjusted models. Specifically, complete household income loss, tertiary/post-secondary education, urban status, levels of concern about COVID-19, and having a friend or family member affected by COVID-19 show significant associations with food insecurity

## Discussion

Aside from the loss of life due to COVID-19, research on the effects of COVID-19 has shown that one of the primary effects of COVID-19 during the heightened levels of the pandemic was the loss of jobs and income due to lockdown measures imposed by national and regional governments as a means of controlling the pandemic [2, 15, 31, 48]. This resulted in untold hardship among vulnerable populations, such as women. Food insecurity increased because of these lockdown measures.

In our study of food insecurity among women in Burkina Faso, we found that 16.97% of the women who were surveyed during the period reported food insecurity, and the total proportion of women who lost partial and complete household income was approximately 74.05%. This finding is not surprising, as authors such as Grugel et al. [16] have argued that COVID-19 and lockdown measures had a significant effect on women in the Global South, particularly women in sub-Saharan African countries. This finding is also consistent with the work of Nwafor [30], who reported that the closing down of schools, markets, and borders; the ban on mass gatherings and travels; and quarantine in Burkina Faso had a consequential effect on women’s income, rendering them vulnerable to food insecurity [30].

Income is the key to sustaining livelihoods in any economy. The loss of income among women significantly affected their food security. We found that women who reported partial income loss were 1.82 times more likely to report food insecurity than women who lost no income (Table 2), after adjusting for other covariates. Similarly, women who reported complete income loss were 5.16 times more likely to report food insecurity than those who lost no income, and this result remained statistically significant after adjusting for other covariates. This result is similar to those of other studies that reported that income loss was a barrier to food security during the COVID-19 lockdown [, 20, 29]. For example, in Kenya and Uganda, one of the devastating effects of the COVID-19 pandemic on households was a reduction in income. Income loss resulted in food insecurity and low dietary quality, as people could no longer have access to the market or purchase food [23].

The findings of our study also suggest that higher socioeconomic status is associated with a lower likelihood of food insecurity among women during the COVID-19 pandemic. Vulnerability to food insecurity was also observed among women who had never attended school compared with educated women. Women who had some formal education were less likely to report food insecurity than women who had never attended school. More significantly, women with tertiary or post-secondary education were 70% (AOR = 0.3) less likely to report food insecurity than those who had never attended school. The effect of education on food insecurity has been well documented in the literature [4, 45]. Educational attainment has been found to provide a safety net for women. Education provides an avenue for gainful employment, particularly in the formal sector, whereas women who have never attended school are more likely to be unemployed or employed in the informal sector. During the COVID-19 lockdown, informal activities were disrupted, which may have affected the income of those who never attended school. However, women employed in the formal sector (due to their higher educational status) were more likely to continue earning income despite the restrictions. According to a United Nations report [41], ‘‘the economic impacts are felt poignantly by women and girls who are often employed in the informal sector, generally earning less, saving less, and holding insecure jobs or living close to poverty.”

In our analysis, divorced or separated women were more likely to experience food insecurity than single women, with an adjusted odds ratio (AOR) of 1.96 (95% CI: 0.60–6.31). The high AOR for divorced or separated women suggests that this group may face unique challenges that increase their risk of food insecurity, such as reduced economic support or increased caregiving responsibilities. However, owing to the small sample size of these groups, as shown in Table 1, the confidence interval had a broad range, making the estimates less precise. Further research with larger sample sizes or studies specifically targeting divorced or separated women could provide more precise estimates and a better understanding of the factors contributing to their food insecurity.

The rural-urban divide was also associated with self-reported food security among women. After adjusting for other covariates, women living in urban areas were 41% (AOR = 0.59) less likely to be food insecure than their rural counterparts. Some reasons can be attributed to the differences in the level of food insecurity between rural and urban women. Although lockdown measures disrupted economic activities in urban areas compared with those in rural areas, and hence, the odds of experiencing food insecurity should have been greater among urban women than among rural women, some reports on COVID-19 safety net programs provided by the Burkinabe government appear to have benefited urban dwellers more than rural dwellers [33, 39]. This might have provided a buffer against the odds of experiencing a higher level of food insecurity among urban women relative to rural women. For example, Pambe et al. [33] stated that the government took over the operating costs of the people working in the market and provided subsidies for access to basic services, namely water and electricity. However, this largely appeared to have benefited urban residents compared to their rural counterparts. Additionally, disruption to the operation of smallholder farmers [16] may have increased the likelihood of rural women experiencing hunger compared with that of urban women.

Furthermore, we also found that women who were concerned about the spread of COVID-19 in their community were significantly more likely to experience food insecurity than women who were not concerned. More importantly, we found that women who reported that a friend or family member had COVID-19 were approximately 11 times more likely to have experienced food insecurity than women who did not have a family member or friend infected with COVID. This result may support the argument of Doss et al. [9] and the WHO [48] that the COVID-19 pandemic increased the role of women as primary care givers foregoing their jobs to cater to a family member who was infected leading to income loss and the likelihood of experiencing food insecurity.

Previous pandemics, such as the outbreak of Ebola in West Africa and the HIV pandemic, have been shown to increase the vulnerability of women to food insecurity [1, 24, 27, 35, 40]. Indeed, Doss et al. [9] argued that there is little to no policy that seeks to protect the interests of women during global health pandemics. For example, during the 2014–2016 Ebola disease outbreak in West Africa, the restrictions imposed on the movement of goods and people affected women’s ability to trade, cultivate their lands, and engage in other informal activities. These restrictions led to the loss of income for most women who could not pay back loans taken for businesses [5, 25]. Overall, our findings support the argument that pandemics and disease outbreaks render vulnerable groups poorer and food insecure, as most vulnerable people lose their livelihood and are unable to buy food [3, 17].

Although this study revealed that income loss significantly impacted women’s food security during the pandemic, we did not find a statistically significant increase in the severity of experiencing hunger due to COVID-19 restrictions compared with pre-pandemic period (Table 3). This lack of significance persisted across multiple sociodemographic factors, including household income loss, age, marital status, education level, urban status, and concern about COVID-19. A possible explanation for this result is that while COVID-19 restrictions may have impacted women’s income levels and disrupted food systems and access, it is possible that pre-existing food insecurity conditions in the region mediated their effects. Given Burkina Faso’s long-standing economic and geopolitical challenges, such as high poverty rates and widespread food insecurity [13, 42], it is plausible that the pandemic intensified these pre-existing vulnerabilities without significantly altering them, as our analysis clearly demonstrates. Thus, many women may have already experienced food insecurity prior to COVID-19, making it difficult to isolate the impact of the pandemic restrictions on worsening hunger among women. This constitutes a major limitation in our study in that we are not able to establish an increase in the severity of food insecurity among women due to COVID-19 restrictions compared with the pre-COVID-19 period. Thus, while the preceding discussion has established statistically significant evidence of women experiencing food insecurity due to COVID-19 restrictions, our study did not find statistically significant evidence of an increase in the severity of food insecurity when comparing their experiences before and during COVID-19.

Another limitation of our study is that our main outcome variable (household income) was measured at the household level, whereas the other variables were measured at the individual level. These results may have biased our estimates, but we also believe that women contribute significantly to household income, particularly regarding feeding. Second, because the COVID-19 survey data are self-reported and cross-sectional, they are subject to the issues inherent in self-reported data, including recall bias for some of the responses and difficulty in measuring changes in the population, which does not allow for causality exploration. Finally, we also want to acknowledge that our study is not a comparative assessment of food insecurity among men and women. The PMA datasets used for this study focused on women. Hence, we are unable to make these assessments directly from the results of our work.

Despite these limitations, our study is very significant in terms of the population and location. Although there is a growing body of literature on the effects of COVID-19 on food insecurity, relatively few studies have focused on Sub-Saharan Africa, especially on women [2, 11, 20]. Our study adds to this body of knowledge and specifically focuses on food insecurity among women. None of the food insecurity papers on COVID-19 in the sub-region has focused specifically on women. Our study is therefore unique in exploring how women have been affected by food security due to COVID-19. Furthermore, the dataset used in this study is very reliable for exploring the effects of the COVID-19 pandemic on women. PMA surveys are checked for data errors and the accuracy of data reporting by employing different validation and reliability measures to ensure data accuracy [50] (Zimmerman et al. 2017). Hence, our study provides an accurate representation of women’s food insecurity due to COVID-19 restrictions.

## Conclusion

In conclusion, our study offers insight into the significant impact of the COVID-19 epidemic on food vulnerability among women in Burkina Faso. The research reveals a multifaceted relationship between the pandemic, income loss, and eventual food insecurity, highlighting the vulnerability of women in the face of such crises. In parallel with previous pandemics such as Ebola and HIV, our study underscores the recurrent theme of women’s vulnerability to food insecurity during global health crises.

## Data Availability

No datasets were generated or analysed during the current study.
